# Conceptual breakthroughs of the long noncoding RNA functional system and its endogenous regulatory role in the cancerous regime

**DOI:** 10.37349/etat.2024.00211

**Published:** 2024-02-27

**Authors:** Anyou Wang

**Affiliations:** University of Milan, Italy; Istituto Nazionale Tumori-IRCCS-Fondazione G. Pascale, Italy; Feinstone Center for Genomic Research, University of Memphis, Memphis, TN 38152, USA

**Keywords:** Big data, long noncoding RNA, noncoding RNA, cancer, function, endogenous, regulatory role

## Abstract

Long noncoding RNAs (lncRNAs) derived from noncoding regions in the human genome were once regarded as junks with no biological significance, but recent studies have shown that these molecules are highly functional, prompting an explosion of studies on their biology. However, these recent efforts have only begun to recognize the biological significance of a small fraction (< 1%) of the lncRNAs. The basic concept of these lncRNA functions remains controversial. This controversy arises primarily from conventional biased observations based on limited datasets. Fortunately, emerging big data provides a promising path to circumvent conventional bias to understand an unbiased big picture of lncRNA biology and advance the fundamental principles of lncRNA biology. This review focuses on big data studies that break through the critical concepts of the lncRNA functional system and its endogenous regulatory roles in all cancers. lncRNAs have unique functional systems distinct from proteins, such as transcriptional initiation and regulation, and they abundantly interact with mitochondria and consume less energy. lncRNAs, rather than proteins as traditionally thought, function as the most critical endogenous regulators of all cancers. lncRNAs regulate the cancer regulatory regime by governing the endogenous regulatory network of all cancers. This is accomplished by dominating the regulatory network module and serving as a key hub and top inducer. These critical conceptual breakthroughs lay a blueprint for a comprehensive functional picture of the human genome. They also lay a blueprint for combating human diseases that are regulated by lncRNAs.

## Introduction

Noncoding regions occupy more than 98% of the human genome, and almost all of them (> 93%) are actively transcribed [1–3]. These transcripts are dominated by long noncoding RNAs (lncRNAs), including lncRNAs, antisense RNAs, and pseudogenes in the present review. The transcription of lncRNAs requires a large amount of energy in the human genome. Theoretically, energy consumption generates a function to fit the low-energy law in a stable physical system. Therefore, all energy-consuming lncRNAs hypothetically carry a certain degree of biological function under given conditions, although most of these functions remain unknown. Understanding the functions of these lncRNAs is critical to understanding their functions in the big picture of the human genome.

Recent evidence suggests that lncRNAs can function as functional molecules, but a real breakthrough came with the recognition that lncRNAs play an influential role in processes such as growth and metabolism [4–7]. Since the breakthrough discovery of lncRNA functions, studies on lncRNA functions have exponentially increased [4–13], attempting to address basic concepts of their biology, such as transcription initiation and regulatory systems [14]. These lncRNA functional studies have conventionally adopted the conceptual framework of protein-based functional systems, which has provided exciting data to provide a preliminary picture of lncRNA function [14]. For example, protein-based polymerase II (Pol II) has been recognized as a primary enzyme that initiates lncRNA transcription [14]. Identification of lncRNAs has also been based on the concept of protein identification, using promoters, start codons, poly(A) tails, Pol II, and DNA conservation [15]. The GENCODE project V35 combined both messenger RNA (mRNA) concepts and sequencing approaches to identify 40,702 lncRNAs, which merged long intergenic noncoding RNAs (lincRNAs) and antisense RNAs [16]. The Functional ANnoTation Of the Mammalian genome (FANTOM) project employed the 5’ strategy to capture 5’ mRNA caps and identify 19,175 lncRNAs [17]. However, these lncRNA functional studies were conducted using conventional approaches that rely on individual experiments and limited datasets. This leads to unavoidably biased observations specific to biological conditions such as tissue types and genetic and epigenetic backgrounds. For example, 78% of lncRNAs collected by the GENCODE project V35 [16] and FANTOM project [17] are condition-dependent [1]. These condition-specific studies are unlikely to generate endogenous lncRNA patterns for understanding the general principles of lncRNA biology.

The concept of condition-dependent lncRNA function has condition-specific implications. It is not surprising that the roles of lncRNAs in cancer are tissue-specific, and lncRNAs have been recognized as secondary factors in tumorigenesis. Proteins are considered the most critical factors that regulate cancer progression [18–26]. Intense studies on proteins have generated a wealth of useful data for clinical treatment to extend the life span of cancer patients; however, these conventional studies have failed to uncover the endogenous mechanism of a common regulatory regime shared across all cancer types. The mechanisms underlying specific cancer types and subtypes have been emphasized in cancer research and therapy.

Fortunately, recent big data studies have significantly advanced our understanding of lncRNA functions and created a conceptual breakthrough in the lncRNA functional system [1, 27]. Analysis of multiple large datasets has demonstrated that lncRNAs are evolutionary drivers of animal lifespan across the animal kingdom by lowering energy consumption [28]. This explains why the human genome requires 98% of noncoding regions that perform broader functions than previously thought with low energy. Big data studies have further developed novel computational algorithms that can find endogenous regulatory networks and patterns hidden in heterogeneous human genome data across various biological conditions [1, 27], and discovered that lncRNAs possess a distinctive functional system that is distinct from that of proteins and is endogenous in the human genome independent of conditions [1]. For example, lncRNA transcription initiation and regulation are distinct from the mRNA-protein-based Pol system, and they are endogenous in the human genome across various physiological states measured by experimental designs deposited in the Sequence Read Archive (SRA) [29] database, which will be discussed in detail in this review.

This conceptual breakthrough in endogenous lncRNA functional systems lays a fundamental foundation for understanding the endogenous roles of lncRNAs in various physiological states such as tumorigenesis. As expected, another big data study revealed that lncRNAs serve as critical regulators in cancers and are endogenous in the cancerous region across all cancer types measured today by The Cancer Genome Atlas (TCGA) [30], whereas proteins only function under normal conditions [31].

This novel lncRNA functional system and its endogenous regulatory roles in the cancerous region have established a new conceptual framework for future functional studies of noncoding regions that occupy most of the human genome. Understanding these breakthrough concepts will help shorten the time frame required for hunting lncRNA functions. This review discusses the details of these discoveries and focuses on the conceptual breakthroughs. As this is not a comprehensive review, it does not contain basic information about lncRNAs. Readers interested in lncRNA comprehensive review of lncRNA function systems, please refer to recent excellent publications such as “Gene regulation by lncRNAs and its biological functions” by Statello et. al. [32], and “Mechanisms of lncRNA biogenesis as revealed by nascent transcriptomics” by Nojima and Proudfoot [14].

## Big data approach

Before discussing biological concepts, it is necessary to briefly introduce the basic elements of big data studies. Massive data study relies on two key elements: (A) a massive, heterogeneous dataset that is large and heterogeneous enough to represent all conditions of a biological state and (B) a computational algorithm to generate unbiased results from a very large data set.

In contrast to conventional methods, which face challenges when dealing with heterogeneous data, the massive data approach welcomes heterogeneous data. Indeed, more heterogeneous data are better for big data analysis, in which more heterogeneous data help to generate more robust endogenous patterns. Therefore, the big data approach generally requires sufficiently large samples to represent all the conditions of a biological state [1]. For example, all human RNA sequencing (RNA-seq) samples from the SRA database that contains samples from almost all experimental conditions should be a substantial dataset representing all humans. In addition, all RNA-seq samples collected by TCGA from samples of the 36 most common types of cancer should constitute one massive dataset for all cancers. Other big data resources have been previously summarized [33].

On the other hand, big data studies require a computation algorithm capable of generating endogenous interactions from heterogeneous data after it has been collected. Although numerous algorithms and platforms have been applied to big biological data studies [34–37], such as artificial neural networks, support vector machines, and decision trees [35], these algorithms mostly perform classification. Conventional network inference algorithms such as C3NET and ARACNe-AP can infer gene interactions [38–41]. However, these conventional software packages suffer from high noise, which can contain more than 90% false positives [27, 41, 42]. In addition, machine learning with graphs [43, 44] has been widely applied to infer interaction networks, but it faces challenges in handling highly heterogeneous biological data with many more variables (genes) than observations (samples).

A novel software called Fast Inferring NETwork (FINET) [27] was developed to infer endogenous interaction networks from highly heterogeneous data. FINET infers any network quickly and accurately and infers endogenous regulatory interactions from highly heterogeneous biological data with > 94% precision as true positives/true positives + false positives. FINET speed and accuracy come from its implementation under fast Julia with stability selection, elastic-net machine learning, and parameter optimization algorithms. In addition to its first accuracy, FINET is user-friendly, with only a single command line to complete all computational processes, and it works in any OS system although FINET was developed under Linux. FINET is a critical tool for uncovering true interactions in big data. Understanding the algorithm helps in its wide applications. This review briefly describes the FINET algorithm and its application.

In a matrix with observations (bio-samples) as rows and variables (genes in biology) as columns, FINET [27] treats each gene as a target (set as *y*) and searches for its regulators from the remaining genes (set as *X*). The target-regulator interaction was inferred using an elastic net model [45].

**Figure:**
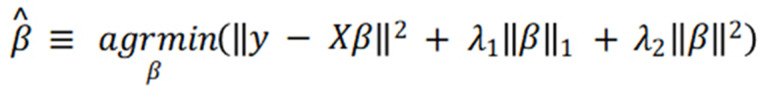


The elastic net is arguably one of the most effective models for gene selection. Despite this, it is likely to produce more than 90% false positives when used alone to infer gene interactions in biology [27].

To reduce the false positives generated by the elastic net model, stability selection has been proposed [42]. Stability selection randomly splits the samples into two groups. When a target-regulator interaction is simultaneously selected in two groups, stability selection treats this interaction as true. Although this stability selection has been proven statistically, it could still contain more than 50% false positives in gene interaction inference because of heterogeneous data [27].

**Figure 1:**
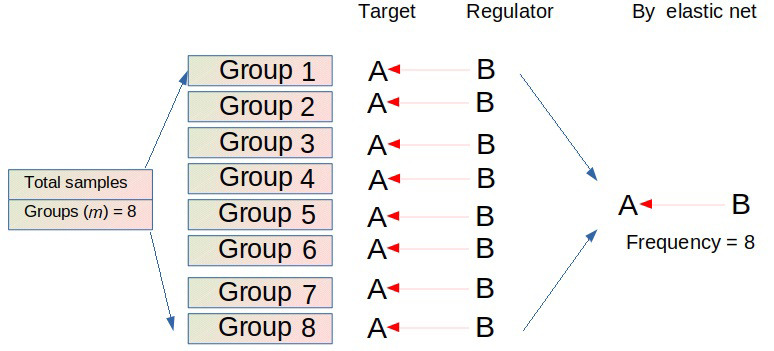
FINET algorithm. FINET splits the total samples into *m* groups (e.g., *m* = 8) and infers gene interactions from each group. If an interaction (e.g., gene B regulating gene A) occurs in each group, this interaction has a perfect frequency (e.g., 8 here). This process iterates *n* times (e.g., *n* = 50). The possibility of finding an interaction that consistently appears in all trials (*m* × *n*) in a large heterogeneous dataset is low. Setting a possibility threshold to filter the low number of possible interactions is a reasonable solution. Therefore, FINET calculates the frequency score by dividing the total frequency by the total number of trials (*m* × *n*). When an interaction has a high frequency score (e.g., frequency score > 0.95), meaning that this interaction appears in most cases (e.g., 380 out of 400 trials here), FINET treats this gene interaction as an endogenous target-regulator pair

To improve the inferring precision and minimize false positive rates, FINET employs the following algorithm: FINET randomly splits the total samples into multiple groups, such as eight groups (*m* = 8), and independently infers gene regulatory interactions from each group using the elastic net (Figure 1). This process iterates *n* times. Interactions with high frequency during these *m* × *n* operations are treated as reliable interactions. Actually, the FINET algorithm filters condition-dependent interactions and retains conditionally independent interactions as endogenous ones.

Based on the algorithm described above, FINET can be widely applied to any type of big data. It has been applied to compute massive heterogeneous data, and its results have been validated [1, 28, 31]. For example, FINET has inferred endogenous regulatory lncRNA networks from all 265k human RNA-seq samples from the SRA database [1] and revealed endogenous lncRNAs from unannotated regions of the human genome [1, 31]. In addition, FINET unearthed an endogenous regulatory network for all cancers based on TCGA data [31]. Moreover, FINET has been applied to identify genome sequence motifs in evolutionary studies of the animal kingdom [28] and severe acute respiratory syndrome coronavirus 2 (SARS-CoV-2) virus [46].

Together, the big data approach provides a promising way to minimize noise and understand the endogenous true picture of heterogeneous big data.

## lncRNA endogenous functional system

In contrast to conventional protein-dominated functional systems, noncoding RNA (ncRNA) functions are thought to be secondary in the human genome. However, a recent big data study revealed that ncRNAs, instead of proteins, drive animal lifespan evolution in the entire animal kingdom [28]. ncRNAs increase their content in animal genomes during evolution and coincide with trimming mitochondrial genome length, which is associated with lower energy consumption. Moreover, more active ncRNAs in the female reproductive system than their male counterparts account for why women outlive men [28]. These results indicate that ncRNAs are crucial functional genes in the two most important traits in humans, including reproduction and longevity. This discovery also emphasizes the biological significance of 98% of noncoding regions in the human genome and suggests that ncRNAs, rather than proteins, carry out most biological functions in the human genome. Understanding the functional system becomes critical for understanding human genome functions. However, the underlying ncRNA functional system remains largely unknown, and it is challenging to explore this complex ncRNA functional system in conventional studies.

A recent big data study was undertaken to capture the big picture of the lncRNA functional system in human genomes [1, 31], in which massive amounts of data were downloaded from the SRA [29] by searching human genomes and RNA sequences without filtering them out based on physiological conditions. This dataset contains all human RNA-seq data deposited in the SRA database, including 265,361 SRA samples under various experimental conditions, such as tissues, cell lines, and physiological conditions. These data are sufficiently comprehensive and heterogeneous to represent all types of conditions. Endogenous lncRNAs, including both annotated and unannotated lncRNAs, in this dataset reflect endogenous lncRNAs in heterogeneous human genomes. The functional characteristics of these endogenous lncRNAs represent the general endogenous traits of lncRNAs in all human genomes. In addition, the big picture derived from these endogenous lncRNAs represents the key biological principles of lncRNAs in all human genomes. As discussed in the following sections, these biological principles form the basis for understanding the functional system of lncRNAs in the human genome.

### Distinctive lncRNA transcription initiation

Although the mechanism of lncRNA transcription initiation remains unclear, it has been assumed that mRNA transcription initiation mechanisms can be adopted for lncRNAs [14, 15, 47]. In the mRNA-coding protein system, histone proteins tightly wrap DNA into a highly condensed chromatin structure containing a series of basic structural units called nucleosomes. Chromatin structure is very stable and prevents DNA from being transcribed into mRNAs. When a pioneer transcription factor binds to chromatin in a gene promoter region, chromatin modification occurs at the initiation region to expose DNA. Transcription factors bind to exposed DNA and recruit Pol II to initiate transcription. Once initiated, transcription can occur bidirectionally, with sense mRNAs and antisense lncRNAs being the major categories of lncRNAs [14].

Chromatin modification is critical for transcription initiation of both lncRNA and coding mRNAs. In yeast, mutating nucleosome chaperones alter the chromatin structure and expose DNA, resulting in Pol II initiation [48]. Loss of chromatin remodelers such as Isw2, which suppresses antisense lncRNA transcription, also generates both coding and lncRNA transcripts [49]. Modifying the chromatin template during transcription can enhance the efficiency of RNA synthesis and pre-mRNA processing [50]. For example, histone H3 lysine 4 trimethylation (H3K4me3) over promoter regions activates transcriptional elongation, enhances capping, and recruits splicing factors from the Pol II complex [51, 52]. H3K4me3 also reactivates multiple rounds of transcription [53]. Similarly, H3K36me3 facilitates efficient elongation, splicing, and 3’ end processing [50].

There are three distinct Pols in the human genome that transcribe ncRNAs: RNA Pol I, Pol II, and Pol III [14]. Pol I transcribes ribosomal RNA (rRNA) and Pol III transcribes smaller, structural ncRNAs, such as transfer RNAs (tRNAs) and 5S rRNA. While Pol I and Pol III transcripts are derived from approximately 30% of the total nuclear transcription, Pol II predominantly works for the remaining 70% of transcriptions, although the transcripts derived from Pol II are generally less stable than those from Pol I and Pol III. Pol II has been characterized as the primary factor transcribing protein-coding genes and lncRNAs [14].

The Pol II complex typically resides in several gene promoters and does not allow the gene to initiate transcription [50]. For most genes, transcription initiation is normally enhanced by enhancers and ancillary regulatory elements, which interact with the gene promoter to form a transcription initiation hub [54]. Therefore, chromatin modifications, Pol II, and enhancers play key roles in gene transcription [15, 47]. Conventionally, this mechanism has been used to explain the initiation of the transcription of both antisense lncRNAs and sense mRNAs.

In a recent big data study, the profiles of histone modifications, Pol II, and enhancers were systematically examined [1]. This big data study investigated the transcription initiation profiles of 14,122 genes for both active protein-coding genes and functional lncRNAs. The initiation profile was analyzed using 780 chromatin immunoprecipitation sequencing (chip-seq) samples, and the top nine factors were measured using Encyclopedia of DNA Elements (ENCODE) [3]. These nine factors included assay for transposase accessible chromatin with high-throughput sequencing (ATAC-seq) for chromatin accessibility, three markers for enhancers [histone H3 lysine 4 monomethylation (H3K4me1), histone H3 lysine 27 acetylation (H3K27ac), and H3K9ac], three markers for promoters [H3K4me3, Pol II subunit alpha (POLR2A), and H3K36me3], and two markers for silencing and tissue specificity (H3K27me3 and H3K9me3). The measurement of these marker profiles varies among tissues and cell lines in the ENCODE project. For unbiased results, this big data study included all measurements conducted by ENCODE, without filtering out any tissues or cell types.

The overall profiling of these protein-coding gene measurements was in agreement with the conventional concept of transcription initiation, as described above, in which all 14,122 gene promoter regions were densely surrounded by the biomarkers of Pol II, enhancer, and chromatin modification. Multiple markers usually bind to a gene promoter simultaneously, with a minimum binding frequency of 75% (10,592 out of 14,122) of any marker binding to a gene promoter.

Unexpectedly, lncRNAs exhibited a marker-binding profile distinct from that of protein-coding genes. First, putative lncRNA promoter regions, defined as 5,000 base pairs (bp) within the transcription start site (TSS), barely exhibited POLR2A binding. Only 12% (a median of 1,668 out of 14,122 lncRNAs) of the lncRNA promoter regions showed POLR2A binding. This indicates that more than 88% of the lncRNAs do not require POLR2A during their initiation. This suggests that Pol II is not a key player in activating lncRNA transcription, as was previously thought. Second, all three enhancer biomarkers, H3K4me1, H3K27ac, and H3K9ac, showed only 16% binding frequency. The low binding frequency (16%) of enhancers might not account for widespread lncRNA transcription initiation, although this 16% was significantly higher than that of POLR2A (12%; Kruskal-Wallis, *P* < 2.2e−16). In addition, binding of the enhancer marker H3K4m1 across the lncRNA promoter regions was significantly higher than that of H3K4me3, a marker for active promoters near the TSS of protein-coding genes. This indicates that enhancers contribute more to lncRNA activation than Pol II. Third, lncRNA promoters carry only limited binding of chromatin modification markers, with a median of 14% (1,990/14,122) of binding sites, suggesting that most lncRNA initiations (more than 86%) do not require protein-based chromatin modifications, similar to protein-coding genes.

Furthermore, the binding distances of these markers to the lncRNA TSS differed from those of protein-coding genes. When the minimum distance from marker binding to TSS was measured, the medians for lncRNAs ranged from 240 bp to 336 bp, while they ranged from 50 bp to 120 bp for protein-coding genes, which were significantly different (*P* < 2.84e−06). This provides another line of evidence that lncRNA initiation is distinct from that of protein-coding genes.

The overall binding frequency of Pol II, enhancers, and chromatin modifications in lncRNA promoters was too low to explain the widespread lncRNA initiation. The binding distances of these biomarkers to the lncRNAs were far from their TSS values. These findings suggest that lncRNAs possess initiation mechanisms distinct from those of proteins. The key factors underlying the activation of most lncRNAs remain to be investigated.

### Primary regulators of lncRNAs: lncRNAs

The current conventional concept of lncRNAs assumes that proteins are primary regulators of lncRNAs [14, 50, 55]. For example, the integrator complex integrator-protein phosphatase 2A complex (INTAC) attenuates lncRNA transcriptional elongation [55]. While these conventional findings offer some explanation for lncRNA processes, such as initiation and attenuation, the exact molecules that play a crucial role in lncRNAs remain unanswered.

Recent big data studies have filled the gap between individual regulators and systems versions of lncRNA regulators [1], providing a big picture of the primary regulators of lncRNAs, in which lncRNAs predominate primary regulator profiling and proteins only serve as secondary regulators. More than 65% of unannotated lncRNA regulators are endogenous lncRNAs [1]. Consistently, annotated lncRNAs also function as the most abundant regulators of annotated lncRNAs [31], suggesting that lncRNAs are the primary regulators of lncRNAs. The self-regulation of lncRNA-lncRNA lays the foundation for the overall functional system of lncRNAs, including their initiation and activation. This self-regulation may be weak under normal conditions, but highly activated under stimulation [31, 56].

In fact, recent advances in technologies examining the high-dimensional structures and expression of RNA also reveal the regulatory roles of RNA in regulating RNA themselves [14, 50]; however, only big data studies can provide a comprehensive picture of the lncRNA-lncRNA regulatory mechanism.

**Figure 2:**
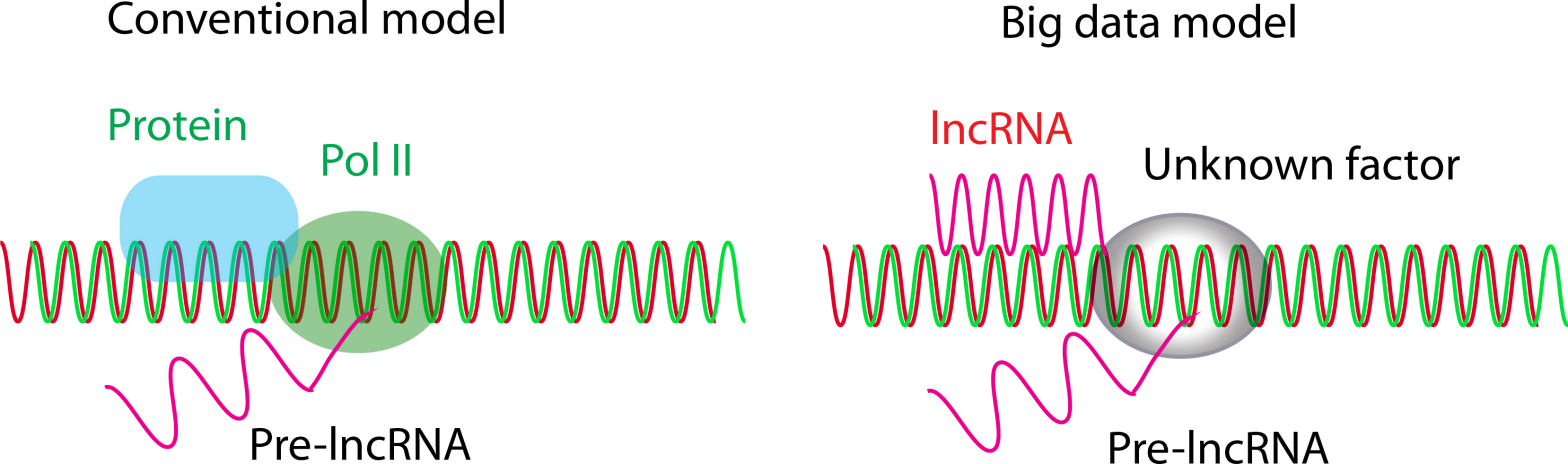
lncRNA initiation model. In the conventional model, lncRNAs are primarily initiated by Pol II and regulated by proteins, while in the big data model, lncRNAs are primarily initiated by unknown factors and regulated by lncRNAs

Together, lncRNAs have a unique functional system distinct from that of mRNA proteins, in which lncRNAs are transcriptionally initiated by unknown initiation factors and are primarily transregulated by lncRNAs from other chromosomes (Figure 2).

According to the same big data study, proteins account for only 22% of lncRNA regulators [1]. This indicates that proteins can only act as secondary regulators of lncRNAs, as opposed to serving as primary regulators, but mitochondrial proteins such as mitochondrially encoded cytochrome C oxidase I (MT-CO1) should not be ignored. These mitochondrial proteins target several lncRNAs. For instance, MT-CO1 regulates more than 400 lncRNA targets [1]. These abundant mitochondrial proteins and their targets in the endogenous lncRNA network suggest that mitochondria play a critical role in regulating lncRNAs and ncRNAs are strongly associated with energy-consuming processes. Consistently, the most recent discovery from big data also uncovered ncRNAs associated with mitochondrial low energy-consuming [28].

### Do lncRNAs target neighboring genes?

Understanding the majority of lncRNA target locations is the first critical step toward understanding their functions and mechanisms. However, the complexity of lncRNA interactions in the human genome makes it challenging to capture a large picture, leading to controversial discussions [57, 58]. Conventional studies have speculated that lncRNAs tend to target their neighboring protein-coding genes [15, 59]; however, recent comprehensive lncRNA networks of both annotated and unannotated lncRNAs based on big data have answered “NO” to this conventional notion [1, 31]. lncRNAs do not primarily regulate their neighboring protein genes or cognate genes via complementary sequences. More than 57% of lncRNAs transregulate their targets across chromosomes [1, 31]. Consistently, the majority of lncRNAs are located in the cytoplasm as transregulators [60].

### Targets of a single lncRNA

In contrast to a single protein that regulates hundreds of targets, a single lncRNA typically regulates a few selected targets. This is the maximum case for 12 proteins and 10 lncRNAs targeted by a single unannotated lncRNA, as revealed by big data [1], suggesting that lncRNAs mediate their targets in a specific and precise manner. Interestingly, most lncRNA targets are proteins (> 55%) [1]. This is consistent with the conventional notion that lncRNAs primarily function as regulators of their target proteins. However, this big data discovery has advanced our understanding of the fundamental drivers of protein-based phenotypes, in which proteins function as molecular phenotypes that are primarily controlled by lncRNAs [1, 31]. Therefore, the observed phenotypes derived from these proteins were mediated by lncRNAs. Therefore, lncRNAs have served as fundamental drivers of protein phenotypes rather than proteins, as is conventionally believed. This parallels recent observations that cancerous phenotypes are expressed by proteins but regulated by lncRNAs [31, 56], as discussed in the cancer section below.

### Broad lncRNA functions

lncRNAs were once thought to be useless junk without functions, but recent studies have recognized them as regulators of several processes [4–7]. A study of big cancer data further demonstrated that lncRNAs are the deadliest regulators of all cancers [56]. However, only a small proportion (< 1%) of lncRNAs has been functionally characterized, and their primary functions in the human genome remain unknown. A recent study on systematically unannotated lncRNAs updated their broad crucial functions involving almost all critical bioprocesses [1], such as DNA replication, nucleic acid metabolism, transcription, RNA processing, cell cycle, and stress response. Therefore, lncRNAs play fundamental roles in the human genome.

## Endogenous regulators of the entire cancerous regime: lncRNAs

**Figure 3:**
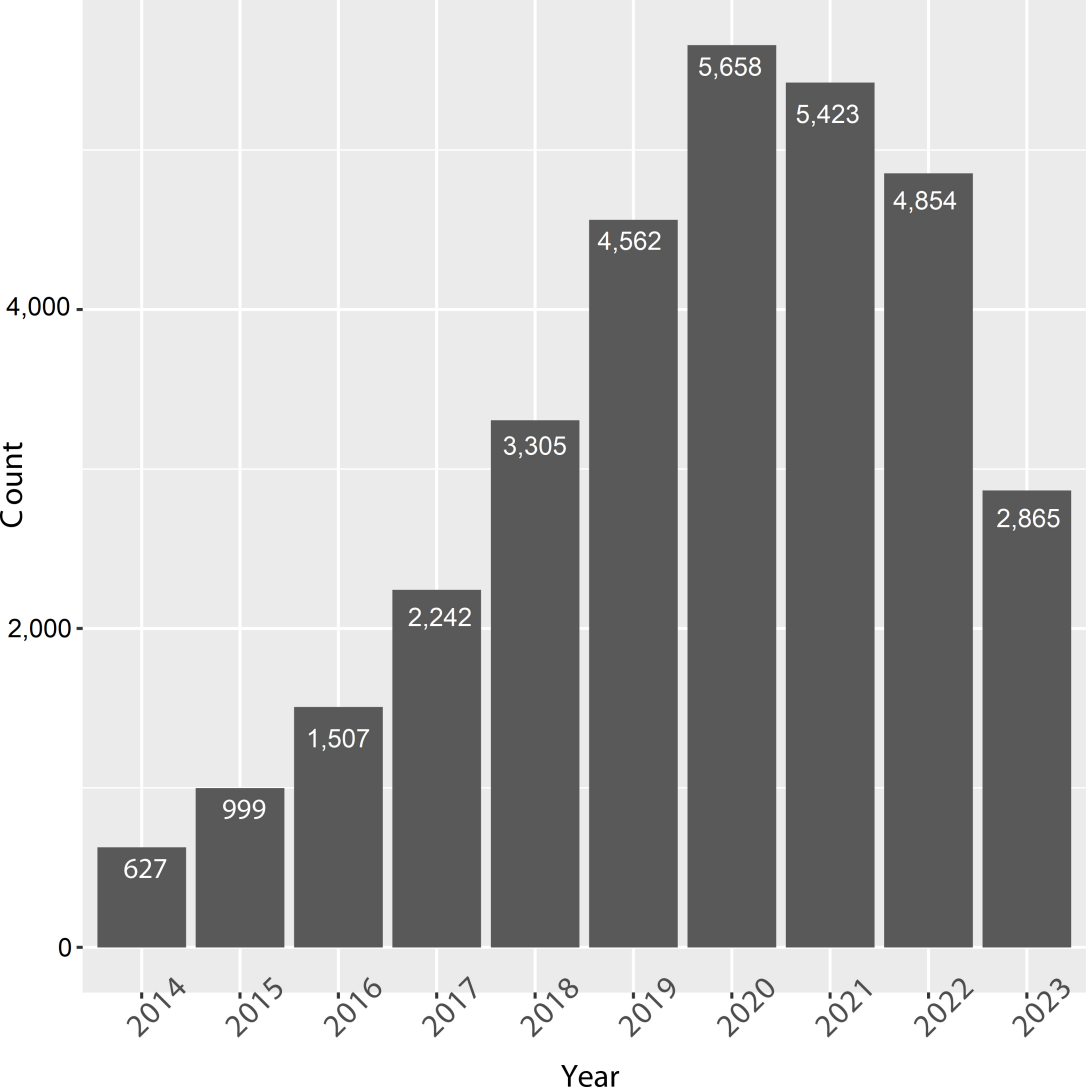
Recent 10-year publications on lncRNAs and cancers. Data was derived from PUBMED by searching “lncRNA and cancers”

Scientific publications regarding lncRNAs and cancers have dramatically increased in recent years, from 627 papers in 2014 to 5,658 papers in 2020 [before the coronavirus disease 2019 (COVID-19) pandemic, Figure 3]. This indicates that the functional role of lncRNAs in cancer has attracted the attention of researchers. However, these studies did not identify the endogenous roles of lncRNAs in all cancers.

All cancers generally result from abnormal genomes evolving into an endogenous regulatory regime that is distinct from that in normal human tissues [61, 62]. Understanding this endogenous regime provides deep insights into the fundamental mechanisms of all cancers, and toward developing a general strategy to combat all cancers. Conventional approaches have been heavily employed to study this regime and to identify endogenous regulators of the cancerous regime [19, 30]. These approaches employ genome sequences, functional genomics, and biochemistry or combinations; however, the complex nature of cancer genomes and heterogeneous cancer data make these approaches ineffective. One of the highly intense study fields employed genome sequencing to identify mutations conserved across all cancers as universal cancer drivers. Projects based on this hypothesis have identified thousands of mutations in both protein-coding regions and ncRNAs in a large number of patient DNA samples from various cancer types [19]; however, no single consensus mutation has been found across all cancer types. Most of these mutations are specific to individual patients. For example, KRAS proto-oncogene, GTPase (*KRAS*) is one of the most mutated genes in lung cancer, but no single *KRAS* mutation is present in more than 40% of patients with lung cancer [19]. *KRAS* mutations are present in less than 2% of cancer types [19]. This indicates that the conventional strategy for identifying conserved mutations is unlikely to identify universal cancer drivers that endogenously regulate all cancer types.

Moreover, conventional biological approaches, such as gene knockout, usually cause transcript compensation [63] and alter whole-genome activation, leading to seriously biased gene regulation. Conventional computational approaches, such as regulatory network inference, usually suffer from high noise with a low accuracy of < 50% [27, 41, 42] when computing heterogeneously complex genome data. Taken together, these results suggest that the current conventional approach faces challenges when revealing endogenous cancerous mechanisms across cancer types.

Uncovering a systemic regulatory network that is endogenous to all cancers provides a foundation for understanding this regulatory regime. A recent big data study utilized FINET [27] to infer an endogenous regulatory network of annotated genes from massive heterogeneous cancer data, including all 11,574 RNA-seq samples and 36 cancer types measured using TCGA. This network discovery has advanced our knowledge of endogenous regulators that modulate this regime, leading to a conceptual breakthrough in cancer biology, as discussed in detail below.

### Dominated lncRNA modules

Theoretically, the network modules execute the primary functions of a network. A network module was constructed using individual components. Therefore, the composition of the module components provides a metric for understanding the module function. The entire endogenous cancerous network was broken down into modules and the module composition for each module was calculated [31]. These modules are then clustered into either protein modules (proteins occupy > 50% of components in a module) or noncoding modules (ncRNAs > 50% of components in a module) [31]. ncRNA modules significantly increased their proportion in the cancerous network to 45.94%, and protein modules decreased to 47.29% compared with that of the normal network (*P* = 0.02963, *χ*^2^ test), in which protein modules accounted for 60.52% of the total network modules, and lncRNA modules only accounted for 28.94% in the normal regime [31]. Notably, 45.94% of the lncRNA modules were derived from annotated lncRNAs measured in this study [31]. In the context of cancer, when unannotated lncRNAs are discovered for a cancer regimen, it is reasonable to assume that lncRNA modules predominate. This is because most ncRNAs in cancers remain unannotated and lncRNAs are key regulators of all cancers [1, 31, 56]. This shift in the network composition to lncRNA modules in the cancer network suggests that lncRNAs drive the cancerous network.

### lncRNAs as the most important rulers in the cancerous regime

**Figure 4:**
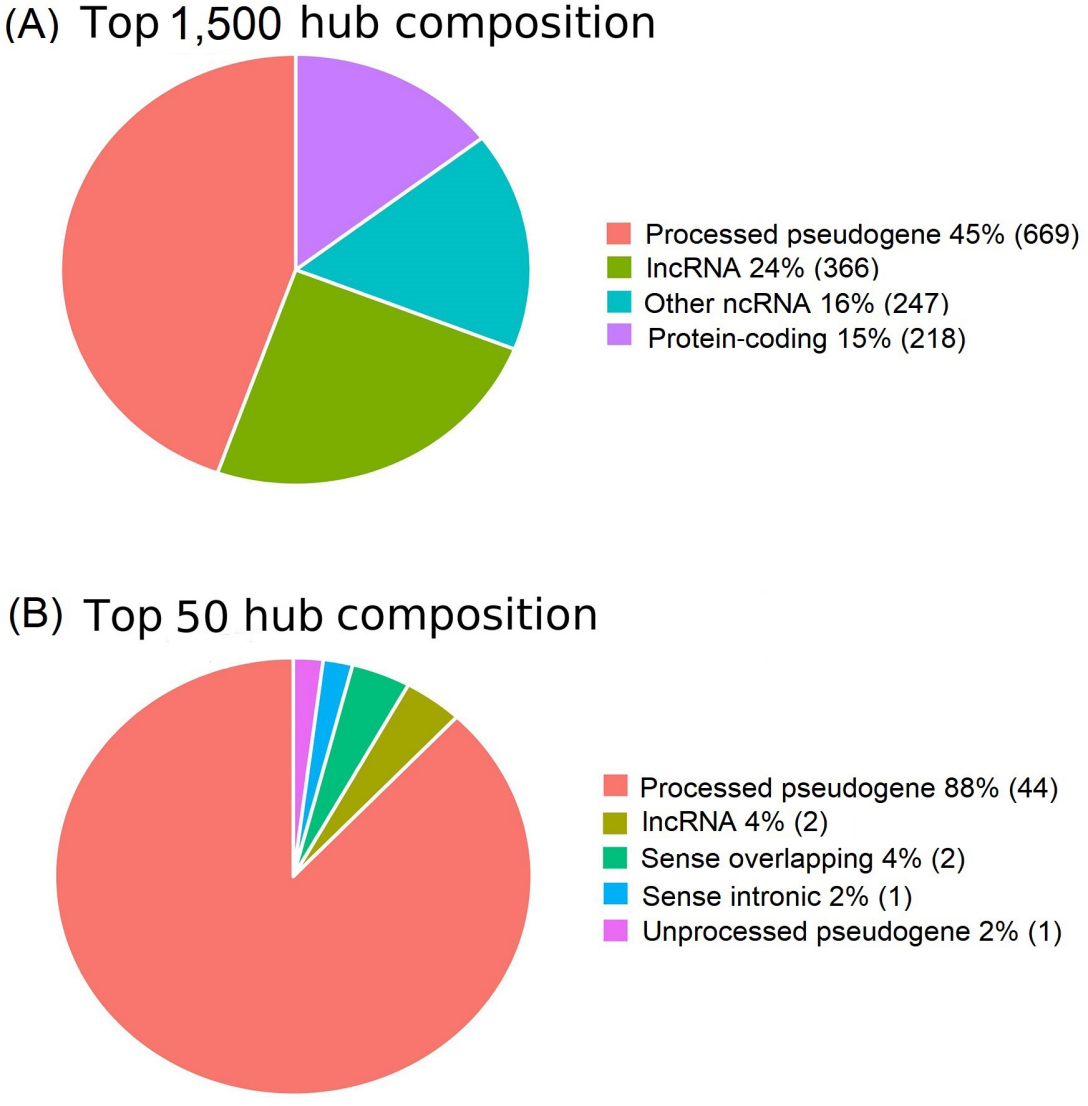
lncRNAs predominate among the top hubs in the cancerous regulatory regime. (A) lncRNAs, especially pseudogenes, predominant in the top 1,500 hubs; (B) lncRNAs predominate among the top 50 hubs

Network hubs are critical network players. lncRNAs serve as the most critical hub in cancerous regions (Figure 4) [31, 64]. Among the top 1,500 hubs, proteins accounted for only 15%, whereas lncRNAs constituted 85% (Figure 4A). In particular, processed pseudogenes account for 45% of the total. Furthermore, lncRNAs accounted for 100% of the top 50 hub profiles (Figure 4B). These results suggested that lncRNAs are the most important regulators of cancer.

**Figure 5:**
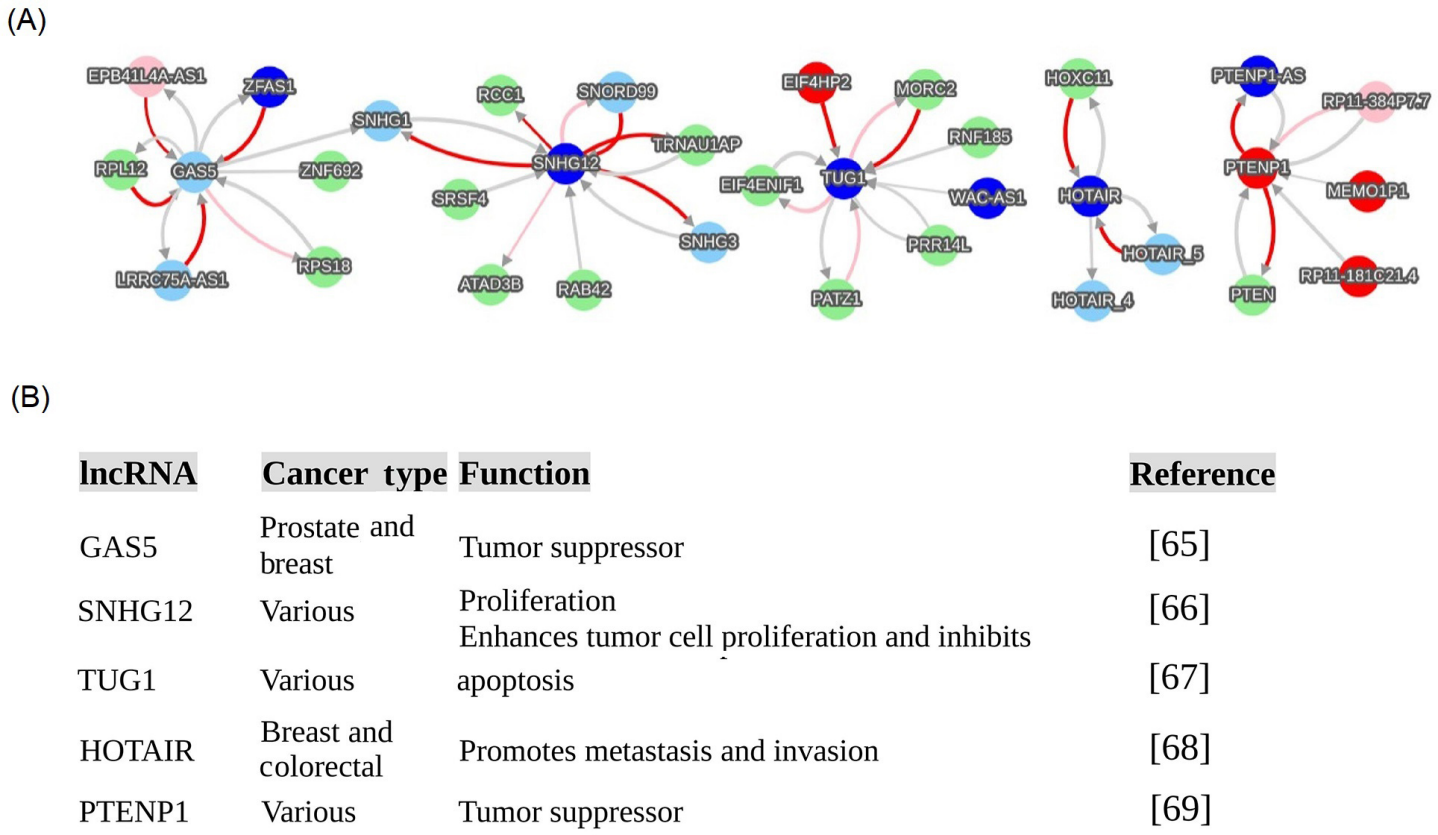
Examples of cancerous lncRNA hubs. These hub interactions were directly extracted from the website of the recent big data discovery (https://combai.org/network/cancer/) via searching five lncRNAs, including GAS5, SNHG12, TUG1, HOTAIR, and PTENP1. (A) Networks of 5 lncRNAs; (B) list of specific functions of 5 lncRNAs in cancers [65–69]. Network annotation: (a) node color denotes gene category, light green, blue, pink, red, and light sky blue respectively denote protein-coding, antisense RNA, lincRNA, processed pseudogene, and other; (b) edge color represents regulation strength: red, pink, and light gray respectively represent strong positive, middle positive, and weak regulation (positive or negative); and (c) edge thickness denotes confidence, thicker, more confident

For detailing lncRNA functions, this review provided five lncRNA functional examples that are regulatory network hubs in cancers (Figure 5): growth arrest specific transcript 5 (GAS5), small nucleolar RNA host gene 12 (SNHG12), taurine up-regulated 1 (TUG1), HOX transcript antisense RNA (HOTAIR), and phosphatase and tensin homolog (PTEN) pseudogene 1 (PTENP1). These networks (Figure 5A) were directly extracted from the cancer database of the endogenous network generated from big data (https://combai.org/network/cancer/) and these functions (Figure 5B) were derived from PUBMED (https://pubmed.ncbi.nlm.nih.gov/). These networks provided a comprehensive functional picture of these five lncRNAs in all cancers, and they are not limited to the known cancer types published in PUBMED (Figure 5B).

One example is the PTENP1 and PTEN interaction, which is found only in the cancer network, but not in the normal regime [31]. This is consistent with experimental observations that have only been reported for cancers [18, 58]. Conventional approaches have only revealed PTENP1 as a regulator of PTEN, but big data has expanded the PTENP1-PTEN interaction to a network module containing several novel PTENP1 interacting partners in the cancer regime, including PTENP1 antisense RNA (PTENP1-AS), RP11-181C21.4, PTENP1-MEMO1P1, and RP11-384P7.7 [31]. This PTENP1-PTEN module is driven by the pseudogene PTENP1 instead of the PTEN protein [31], as conventionally thought. PTENP1 and its partners provide a complete picture of PTENP1’s endogenous regulatory roles in all cancers.

Once labeled, junk pseudogenes have recently been reported to be regulators of cognate genes [58], and their functions are thought to be secondary. Indeed, the pseudogenes discussed above act as the most critical drivers, instead of secondary regulators. This is also supported by system-based validation showing that pseudogenes are the deadliest endogenous regulators of all cancers [56].

### lncRNAs as the top cancer inducers

Cancer inducers play a critical role in cancer development. While proteins work as the top inducers in the normal regime, lncRNAs predominate as the strongest inducers in the cancer regime, including processed pseudogene, antisense RNA, and lincRNA [31]. Moreover, clinical data have shown that lncRNAs are the universal deadliest inducers of all types of cancers [56].

Interestingly, these cancer inducers modulate proteins as their major targets (> 98%) [31]. Instead of acting as cancer drivers, proteins serve as lncRNA targets. Therefore, protein functions are molecular phenotypes fundamentally determined by lncRNAs in cancers. The conventional practice of treating proteins as cancerous drivers and monitoring protein activity to determine their fundamental mechanism is misleading.

### lncRNA local targets in cancers

Generally, the majority of lncRNAs serve as trans-regulators to regulate their targets across chromosomes in healthy tissues [1, 31]; however, in cancerous regions, lncRNAs serve as *cis*-regulators that primarily target local proteins [< 1 mega bp (Mb)] [31]. However, lncRNAs rarely regulate their cognates under both normal and abnormal conditions [31]. Therefore, lncRNA regulation switches from normal trans-regulation to cancerous *cis*-regulation; however, lncRNAs are not cognate regulators.

### lncRNAs biomarkers to detect all cancers

Detecting cancer at the population level is one of the most effective ways to save the lives of cancer patients. Although technological advancements, such as microarray and sequencing, provide rich resources for the development of efficient detection systems, no practical system is available for clinical use. The core challenge in developing such a system is identifying a set of endogenous biomarkers for all cancers. In a recent big data study, lncRNAs have been identified as endogenous cancer biomarkers [56, 64, 70]. Incorporating these lncRNA biomarkers with artificial neural networks can accurately discriminate all cancers with a 96% area under curve (AUC) of a receiver operating characteristic curve (ROC) [64]. This provides a platform for screening for cancers at the population level.

**Figure 6:**
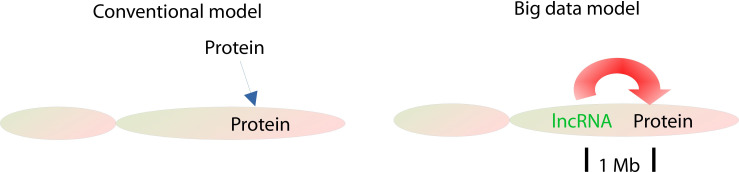
lncRNA working models in cancer cells. In the conventional model, proteins serve as primary regulators and trans-regulate proteins in cancers, but in the big data model, lncRNAs work as the primary regulators and primarily *cis*-regulate the local proteins as their targets within approximately 1 Mb

Therefore, lncRNAs, rather than proteins as conventionally thought, serve as the most important regulators of the tumorous regime and *cis*-regulate their local (1 Mb) protein as their targets (Figure 6).

## Clinical applications of lncRNAs in cancers

With more functions of lncRNAs found in cancers, their clinical applications in cancers have increased linearly every year (Table S1). In 2022, there were six lncRNA clinical applications, and a total of 28 clinical applications were available in the clinicaltrials.gov database. These applications include drug targeting, diagnostic testing, and screening. Interestingly, exosomal lncRNAs have been used in cancer detection. This parallels our recent report showing that ncRNAs and an artificial intelligence (AI) neural network can detect all cancers with 96% of AUC.

## Conclusions

Why the human genome contains 98% noncoding regions remains a mystery, but a recent big data study has dramatically advanced the understanding of its biological significance [28]. ncRNAs work with mitochondria in a low-energy fashion and extend animal lifespan during evolution; they also serve as crucial players in the human reproductive system [28]. Lifespan and reproducibility are the two most important traits in the evolution of any species. Without strong reproduction and long longevity, the species disappears in a long evolutionary process. Humans evolutionarily gain an increasing number of ncRNAs in their genomes to live longer and produce more generations. Assuming that these 98% noncoding regions were converted into protein-coding regions, humans might only survive a week as yeasts, which contain fewer noncoding regions and live only about a week. Therefore, 98% of these noncoding regions are functionally critical for humans. Consistently, recent big data studies have also unearthed ncRNAs as endogenous regulators for all cancers, whereas proteins only work endogenously under normal physiological states [31]. This suggests that under abnormal conditions, human ncRNAs are functionally more important than proteins. Humans live mostly in abnormal environments, such as various diseases, microbial infections, and variable environmental changes. These abnormal factors force humans to gain 98% of noncoding regions that are efficiently transcribed as ncRNAs that perform functions to cope with certain physiological states. Comprehending these ncRNA functions will be a key task in human genome research in the near future.

Comprehending ncRNA functions is challenging for conventional scientists, but big data scientists have taken advantage of the massive amount of available data and have revealed the big picture of human lncRNA functional systems [1]. In contrast to the conventional notion that lncRNAs are the secondary components in protein-based functional systems, the breakthrough discovery emphasized that lncRNAs have a distinctive functional system that is different from protein-based systems, and this system is endogenous to all human genomes and is independent of any condition [1]. This conceptual update of the endogenous lncRNA functional system has established a foundation for comprehending human genome function. For example, in this lncRNA system, lncRNAs have a unique transcription initiation system, although their initiation factors remain elusive. Future research using emerging big data will accelerate the discovery of lncRNA initiation factors. This will open a new avenue for understanding the transcriptional mechanisms of the dark regions in the human genome.

Since ncRNAs function as critical players under abnormal conditions and lncRNAs have their own functional system, it is not surprising for big data studies to uncover ncRNAs, instead of proteins as thought, as endogenous rulers for all cancers [31]. This breaks through the concept of fundamental drivers of all cancers. Future research based on this concept will help to elucidate the universal machinery and mechanisms of all cancers. The application of this novel concept will lead to the creation of a universal strategy for the diagnosis and treatment of all cancers. In particular, the biological functions of the abundant pseudogenes that dominate the cancer regime remain elusive. Elucidating the biological significance of these pseudogenes will be critical in cancer research and clinical applications.

Big data studies in biology are in their infancy, but they will evolve rapidly as emerging big data become available. Future research on big data should focus on both specific topics and big-picture analyses of a broad range of massive data to make conceptual breakthroughs in the general fundamental principles of biology. Embedded computational algorithms that integrate AI with emerging data requirements must be developed. These novel computational algorithms will advance the understanding of the fundamental principles of biology and lead to a revolutionary scientific discovery era.

## Abbreviations

bp: base pair
ENCODE: Encyclopedia of DNA Elements
FINET: FAST Inferring NETwork
GAS5: growth arrest specific transcript 5
H3K27ac: histone H3 lysine 27 acetylation
H3K4me3: histone H3 lysine 4 trimethylation
HOTAIR: HOX transcript antisense RNA
*KRAS*: KRAS proto-oncogene, GTPase
lincRNAs: long intergenic noncoding RNAs
lncRNAs: long noncoding RNAs
Mb: mega base pair
mRNA: messenger RNA
ncRNAs: noncoding RNAs
Pol II: polymerase II
POLR2A: polymerase II subunit alpha
PTEN: phosphatase and tensin homolog
PTENP1: phosphatase and tensin homolog pseudogene 1
RNA-seq: RNA sequencing
SNHG12: small nucleolar RNA host gene 12
SRA: Sequence Read Archive
TCGA: The Cancer Genome Atlas
TSS: transcription start site
TUG1: taurine up-regulated 1

## Supplementary materials

The supplementary material for this article is available at: https://www.explorationpub.com/uploads/Article/file/1002211_sup_1.xlsx.

## Declarations

### Availability of data and materials

The datasets for this study can be found in the Sequence Read Archive (SRA, https://www.ncbi.nlm.nih.gov/sra), The Cancer Genome Atlas (TCGA, https://www.cancer.gov/about-nci/organization/ccg/research/structural-genomics/tcga), and ENCODE project (https://www.encodeproject.org/).

### Copyright

© The Author(s) 2024.

## References

[1] Wang A (2022). Distinctive functional regime of endogenous lncRNAs in dark regions of human genome. Comput Struct Biotechnol J.

[2] The ENCODE Project Consortium (2007). Identification and analysis of functional elements in 1% of the human genome by the ENCODE pilot project. Nature.

[3] The ENCODE Project Consortium (2012). An integrated encyclopedia of DNA elements in the human genome. Nature.

[4] Ramilowski JA, Yip CW, Agrawal S, Chang JC, Ciani Y, Kulakovskiy IV (2020). Functional annotation of human long noncoding RNAs via molecular phenotyping. Genome Res.

[5] Parenteau J, Maignon L, Berthoumieux M, Catala M, Gagnon V, Abou Elela S (2019). Introns are mediators of cell response to starvation. Nature.

[6] Morgan JT, Fink GR, Bartel DP (2019). Excised linear introns regulate growth in yeast. Nature.

[7] Wei LH, Guo JU (2020). Coding functions of “noncoding” RNAs. Science.

[8] Ghafouri-Fard S, Dashti S, Taheri M (2020). The role of long non-coding RNA CASC2 in the carcinogenesis process. Biomed Pharmacother.

[9] Winkle M, El-Daly SM, Fabbri M, Calin GA (2021). Noncoding RNA therapeutics — challenges and potential solutions. Nat Rev Drug Discov.

[10] Abdi E, Latifi-Navid S, Latifi-Navid H (2022). LncRNA polymorphisms and breast cancer risk. Pathol Res Pract.

[11] Huang Z, Zhou JK, Peng Y, He W, Huang C (2020). The role of long noncoding RNAs in hepatocellular carcinoma. Mol Cancer.

[12] Poulet C, Njock MS, Moermans C, Louis E, Louis R, Malaise M (2020). Exosomal long non-coding RNAs in lung diseases. Int J Mol Sci.

[13] Adnane S, Marino A, Leucci E (2022). LncRNAs in human cancers: signal from noise. Trends Cell Biol.

[14] Nojima T, Proudfoot NJ (2022). Mechanisms of lncRNA biogenesis as revealed by nascent transcriptomics. Nat Rev Mol Cell Biol.

[15] Ransohoff JD, Wei Y, Khavari PA (2018). The functions and unique features of long intergenic non-coding RNA. Nat Rev Mol Cell Biol.

[16] Human release 35 (GRCh38.p13) [Internet]. https://www.gencodegenes.org/human/release_35.html.

[17] Hon CC, Ramilowski JA, Harshbarger J, Bertin N, Rackham OJL, Gough J (2017). An atlas of human long non-coding RNAs with accurate 5’ ends. Nature.

[18] Poliseno L, Salmena L, Zhang J, Carver B, Haveman WJ, Pandolfi PP (2010). A coding-independent function of gene and pseudogene mRNAs regulates tumour biology. Nature.

[19] Tate JG, Bamford S, Jubb HC, Sondka Z, Beare DM, Bindal N (2019). COSMIC: the catalogue of somatic mutations in cancer. Nucleic Acids Res.

[20] Buisson R, Langenbucher A, Bowen D, Kwan EE, Benes CH, Zou L (2019). Passenger hotspot mutations in cancer driven by APOBEC3A and mesoscale genomic features. Science.

[21] Bailey MH, Tokheim C, Porta-Pardo E, Sengupta S, Bertrand D, Weerasinghe A (2018). Comprehensive characterization of cancer driver genes and mutations. Cell.

[22] Haffner MC, Zwart W, Roudier MP, True LD, Nelson WG, Epstein JI (2021). Genomic and phenotypic heterogeneity in prostate cancer. Nat Rev Urol.

[23] Hausser J, Alon U (2020). Tumour heterogeneity and the evolutionary trade-offs of cancer. Nat Rev Cancer.

[24] Drosten M, Barbacid M (2020). Targeting the MAPK pathway in KRAS-driven tumors. Cancer Cell.

[25] Hu J, Cao J, Topatana W, Juengpanich S, Li S, Zhang B (2021). Targeting mutant p53 for cancer therapy: direct and indirect strategies. J Hematol Oncol.

[26] Dhanasekaran R, Deutzmann A, Mahauad-Fernandez WD, Hansen AS, Gouw AM, Felsher DW (2022). The *MYC* oncogene — the grand orchestrator of cancer growth and immune evasion. Nat Rev Clin Oncol.

[27] Wang A, Hai R (2020). FINET: fast inferring NETwork. BMC Res Notes.

[28] Wang A Noncoding RNAs evolutionarily extend animal lifespan. https://www.biorxiv.org/content/10.1101/2023.06.09.544283v1.

[29] Sequence Read Archive (SRA) [Internet]. https://www.ncbi.nlm.nih.gov/sra.

[30] Liu J, Lichtenberg T, Hoadley KA, Poisson LM, Lazar AJ, Cherniack AD, Hu H, The Cancer Genome Atlas Research Network (2018). An integrated TCGA pan-cancer clinical data resource to drive high-quality survival outcome analytics. Cell.

[31] Wang A (2022). Noncoding RNAs endogenously rule the cancerous regulatory realm while proteins govern the normal. Comput Struct Biotechnol J.

[32] Statello L, Guo CJ, Chen LL, Huarte M (2021). Gene regulation by long non-coding RNAs and its biological functions. Nat Rev Mol Cell Biol.

[33] Jiang P, Sinha S, Aldape K, Hannenhalli S, Sahinalp C, Ruppin E (2022). Big data in basic and translational cancer research. Nat Rev Cancer.

[34] ElSayed IA, ElDahshan K, Hefny H, ElSayed EK (2021). Big data and its future in computational biology: a literature review. J Comput Sci.

[35] Dall’Alba G, Casa PL, Abreu FP, Notari DL, de Avila E Silva S (2022). A survey of biological data in a big data perspective. Big Data.

[36] Yin Z, Lan H, Tan G, Lu M, Vasilakos AV, Liu W (2017). Computing platforms for big biological data analytics: perspectives and challenges. Comput Struct Biotechnol J.

[37] Seyed Tabib NS, Madgwick M, Sudhakar P, Verstockt B, Korcsmaros T, Vermeire S (2020). Big data in IBD: big progress for clinical practice. Gut.

[38] Altay G, Emmert-Streib F (2010). Inferring the conservative causal core of gene regulatory networks. BMC Syst Biol.

[39] Emmert-Streib F, Glazko GV, Altay G, de Matos Simoes R (2012). Statistical inference and reverse engineering of gene regulatory networks from observational expression data. Front Genet.

[40] Hawe JS, Theis FJ, Heinig M (2019). Inferring interaction networks from multi-omics data. Front Genet.

[41] Marbach D, Costello JC, Küffner R, Vega NM, Prill RJ, Camacho DM, Kellis M, Collins JJ, Stolovitzky G, The DREAM5 Consortium (2012). Wisdom of crowds for robust gene network inference. Nat Methods.

[42] Meinshausen N, Bühlmann P (2010). Stability selection. J R Statist Soc B.

[43] Kipf TN, Welling M Semi-supervised classification with graph convolutional networks. https://arxiv.org/abs/1609.02907.

[44] Hamilton WL, Ying R, Leskovec J Inductive representation learning on large graphs. https://arxiv.org/abs/1706.02216.

[45] Zou H, Hastie T (2005). Regularization and variable selection via the elastic net. J R Statist Soc B.

[46] Wang A Evolutionary trajectory of SARS-CoV-2 genome. https://www.researchsquare.com/article/rs-1009010/v1.

[47] Schlackow M, Nojima T, Gomes T, Dhir A, Carmo-Fonseca M, Proudfoot NJ (2017). Distinctive patterns of transcription and RNA processing for human lincRNAs. Mol Cell.

[48] Duina AA (2011). Histone chaperones Spt6 and FACT: similarities and differences in modes of action at transcribed genes. Genet Res Int.

[49] Whitehouse I, Rando OJ, Delrow J, Tsukiyama T (2007). Chromatin remodelling at promoters suppresses antisense transcription. Nature.

[50] Moore MJ, Proudfoot NJ (2009). Pre-mRNA processing reaches back to transcription and ahead to translation. Cell.

[51] Li B, Carey M, Workman JL (2007). The role of chromatin during transcription. Cell.

[52] Sims RJ III, Millhouse S, Chen CF, Lewis BA, Erdjument-Bromage H, Tempst P (2007). Recognition of trimethylated histone H3 lysine 4 facilitates the recruitment of transcription postinitiation factors and pre-mRNA splicing. Mol Cell.

[53] Ng HH, Robert F, Young RA, Struhl K (2003). Targeted recruitment of Set1 histone methylase by elongating Pol II provides a localized mark and memory of recent transcriptional activity. Mol Cell.

[54] Lim B, Levine MS (2021). Enhancer-promoter communication: hubs or loops?. Curr Opin Genet Dev.

[55] Zheng H, Qi Y, Hu S, Cao X, Xu C, Yin Z (2020). Identification of Integrator-PP2A complex (INTAC), an RNA polymerase II phosphatase. Science.

[56] Wang A, Hai R (2021). Noncoding RNAs serve as the deadliest universal regulators of all cancers. Cancer Genomics Proteomics.

[57] Lee JT (2012). Epigenetic regulation by long noncoding RNAs. Science.

[58] Tay Y, Rinn J, Pandolfi PP (2014). The multilayered complexity of ceRNA crosstalk and competition. Nature.

[59] Cabili MN, Trapnell C, Goff L, Koziol M, Tazon-Vega B, Regev A (2011). Integrative annotation of human large intergenic noncoding RNAs reveals global properties and specific subclasses. Genes Dev.

[60] Carlevaro-Fita J, Rahim A, Guigó R, Vardy LA, Johnson R (2016). Cytoplasmic long noncoding RNAs are frequently bound to and degraded at ribosomes in human cells. RNA.

[61] Calabrese C, Davidson NR, Demircioğlu D, Fonseca NA, He Y, Kahles A, Brazma A, Brooks AN, Göke J, Rätsch G, Schwarz RF, Stegle O, PCAWG Transcriptome Core Group, PCAWG Transcriptome Working Group, PCAWG Consortium (2020). Genomic basis for RNA alterations in cancer. Nature.

[62] van de Haar J, Canisius S, Yu MK, Voest EE, Wessels LFA, Ideker T (2019). Identifying epistasis in cancer genomes: a delicate affair. Cell.

[63] El-Brolosy MA, Kontarakis Z, Rossi A, Kuenne C, Günther S, Fukuda N (2019). Genetic compensation triggered by mutant mRNA degradation. Nature.

[64] Wang A, Hai R, Rider PJ, He Q (2022). Noncoding RNAs and deep learning neural network discriminate multi-cancer types. Cancers (Basel).

[65] Ma C, Shi X, Zhu Q, Li Q, Liu Y, Yao Y (2016). The growth arrest-specific transcript 5 (GAS5): a pivotal tumor suppressor long noncoding RNA in human cancers. Tumour Biol.

[66] Tamang S, Acharya V, Roy D, Sharma R, Aryaa A, Sharma U (2019). SNHG12: an lncRNA as a potential therapeutic target and biomarker for human cancer. Front Oncol.

[67] Farzaneh M, Ghasemian M, Ghaedrahmati F, Poodineh J, Najafi S, Masoodi T (2022). Functional roles of lncRNA-TUG1 in hepatocellular carcinoma. Life Sci.

[68] Rajagopal T, Talluri S, Akshaya RL, Dunna NR (2020). HOTAIR lncRNA: a novel oncogenic propellant in human cancer. Clin Chim Acta.

[69] Haddadi N, Lin Y, Travis G, Simpson AM, Nassif NT, McGowan EM (2018). PTEN/PTENP1: ‘regulating the regulator of RTK-dependent PI3K/Akt signalling’, new targets for cancer therapy. Mol Cancer.

[70] Kim T (2023). Nucleic acids in cancer diagnosis and therapy. Cancers (Basel).

